# 1,800 MHz Radiofrequency Electromagnetic Irradiation Impairs Neurite Outgrowth With a Decrease in Rap1-GTP in Primary Mouse Hippocampal Neurons and Neuro2a Cells

**DOI:** 10.3389/fpubh.2021.771508

**Published:** 2021-11-22

**Authors:** Yanqi Li, Ping Deng, Chunhai Chen, Qinlong Ma, Huifeng Pi, Mindi He, Yonghui Lu, Peng Gao, Chao Zhou, Zhixin He, Yanwen Zhang, Zhengping Yu, Lei Zhang

**Affiliations:** Key Laboratory of Medical Protection for Electromagnetic Radiation, Department of Occupational Health, Ministry of Education, Third Military Medical University, Chongqing, China

**Keywords:** radiofrequency electromagnetic radiation, neurite outgrowth, Rap1, Rap1-GTP, Neuro2a cell, primary mouse hippocampal neurons

## Abstract

**Background:** With the global popularity of communication devices such as mobile phones, there are increasing concerns regarding the effect of radiofrequency electromagnetic radiation (RF-EMR) on the brain, one of the most important organs sensitive to RF-EMR exposure at 1,800 MHz. However, the effects of RF-EMR exposure on neuronal cells are unclear. Neurite outgrowth plays a critical role in brain development, therefore, determining the effects of 1,800 MHz RF-EMR exposure on neurite outgrowth is important for exploring its effects on brain development.

**Objectives:** We aimed to investigate the effects of 1,800 MHz RF-EMR exposure for 48 h on neurite outgrowth in neuronal cells and to explore the associated role of the Rap1 signaling pathway.

**Material and Methods:** Primary hippocampal neurons from C57BL/6 mice and Neuro2a cells were exposed to 1,800 MHz RF-EMR at a specific absorption rate (SAR) value of 4 W/kg for 48 h. CCK-8 assays were used to determine the cell viability after 24, 48, and 72 h of irradiation. Neurite outgrowth of primary hippocampal neurons (DIV 2) and Neuro2a cells was observed with a 20 × optical microscope and recognized by ImageJ software. Rap1a and Rap1b gene expressions were detected by real-time quantitative PCR. Rap1, Rap1a, Rap1b, Rap1GAP, and p-MEK1/2 protein expressions were detected by western blot. Rap1-GTP expression was detected by immunoprecipitation. The role of Rap1-GTP was assessed by transfecting a constitutively active mutant plasmid (Rap1-Gly_Val-GFP) into Neuro2a cells.

**Results:** Exposure to 1,800 MHz RF-EMR for 24, 48, and 72 h at 4 W/kg did not influence cell viability. The neurite length, primary and secondary neurite numbers, and branch points of primary mouse hippocampal neurons were significantly impaired by 48-h RF-EMR exposure. The neurite-bearing cell percentage and neurite length of Neuro2a cells were also inhibited by 48-h RF-EMR exposure. Rap1 activity was inhibited by 48-h RF-EMR with no detectable alteration in either gene or protein expression of Rap1. The protein expression of Rap1GAP increased after 48-h RF-EMR exposure, while the expression of p-MEK1/2 protein decreased. Overexpression of constitutively active Rap1 reversed the decrease in Rap1-GTP and the neurite outgrowth impairment in Neuro2a cells induced by 1,800 MHz RF-EMR exposure for 48 h.

**Conclusion:** Rap1 activity and related signaling pathways are involved in the disturbance of neurite outgrowth induced by 48-h 1,800 MHz RF-EMR exposure. The effects of RF-EMR exposure on neuronal development in infants and children deserve greater focus.

## Introduction

The increasing demand for communication technology in modern society has led to an increased use of radiofrequency electromagnetic radiation (RF-EMR) (1). The popularity of mobile phone communication is expanding rapidly. The proportion of 13-year-old teenagers using mobile phones has reached 90% in Korea (2–4). With the use of radio equipment for monitoring and receiving information in neonatal intensive care units, infants are also becoming increasingly exposed to RF-EMR. Thus, the impact of radio frequency devices on infants, children, and adolescents has attracted global attention.

The brain is known to be one of the most sensitive organs to RF-EMR exposure. Neuronal and cognitive functions are found to be influenced by RF-EMR exposure. Studies have indicated that teenagers using mobile phones and thus receiving RF-EMR exposure may experience memory and attention loss, learning and cognitive impairment, increased irritability, sleep problems, increased sensitivity to stress, and increased risk of seizures (5). The use of mobile phones by teenagers has been shown to be related to emotional and behavioral disorders, while reducing mobile phone use can improve children's cognitive ability (6). Our previous epidemiological studies also showed that inattention and fatigue were associated with mobile phone use in children and adolescents (7, 8), Hippocampal tissues relates to learning and memory, is one of the sensitive targets of RF-EMR. Primary hippocampal neurons are classical model cells to evaluate neurite outgrowth. The assessments of average neurite length, number of primary and secondary branches of primary hippocampal neurons were applied in many other studies (9, 10).

As an important functional executor in brain, neuronal cells have attracted the greatest attention in the study of the effects and mechanism of RF-EMR exposure. Delivery of RF-EMR exposure for 24 h can cause marked cell death in rat cortical neurons (11). There is also evidence that the electrical activity of cultured cortical neurons decreases during RF-EMR exposure (12). Furthermore, RF-EMR exposure inhibits the length and number of axon branches of cortical neurons (13). The activity and density of mature spines in primary hippocampal neurons decrease significantly when exposed to 2.4 W/kg RF-EMR, and the average length of dendrites per neuron is also reduced (14). We also showed that RF-EMR exposure impairs neurite outgrowth in neural stem cell-derived neurons (15, 16). However, a study using a pulsed radiofrequency electromagnetic field reported that it can potentiate neurite outgrowth in the dopaminergic MN9D cell line (17). The influence of RF-EMR exposure on neuronal cells remains controversial, but it is generally agreed that neuronal cells, specifically neurite outgrowth, are sensitive to RF-EMR exposure (18, 19). Therefore, it is important to elucidate the effect of RF-EMR exposure on neurite outgrowth.

Rap1 protein, a member of the RAS family, interacts with cAMP, calcium and other second messengers, turning extracellular stimulation into intracellular signals (20). The Rap1 protein acts as a molecular switch by cycling between two states (an inactive GDP-binding form and active GTP-binding form). These modifications are strictly controlled by guanine nucleotide exchange factors (GEFs) and GTPase activating proteins (GAPs) (21). Recent research further confirmed that Rap1 can affect the rearrangement of the cytoskeleton and promote neurite outgrowth and dendritic spine formation in the brain (22). Genetic interaction analysis showed that the repulsive guidance function mediated by the Sema-1a/PlexA signaling pathway, which contributes to axon growth and guidance, was regulated by Rap1 (23). In newborn neurons, Rap1 is a critical regulator in the formation of axons, leading and maintaining the radial migration of neuronal processes (24). Rap1-GTP, the active form of Rap1, is considered to affect the growth and differentiation of neuronal cells (25, 26). Rap1GAP, hydrolyzing the active form of Rap1, is degraded by the ubiquitin proteasome system (27). In the process of angiotensin II-stimulated neurite outgrowth in NG108-15 cells, the Rap1/B-Raf signal cascade activates Mitogen-Activated Protein Kinase Kinase (MEK) and induces sustained activation of p42/p44 Mitogen-Activated Protein Kinase (MAPK) and finally neurite outgrowth (28, 29). Although the role of Rap1 in neurite outgrowth has been widely studied, whether Rap1 is involved in neurite outgrowth regulation when neuronal cells are exposed to RF-EMR remains unclear.

The purpose of this study was to investigate the effect of 1,800 MHz RF-EMR exposure on neurite outgrowth in primary mouse hippocampal neurons and Neuro2a cells and to further explore the role of Rap1 in this process.

## Results

### 48-H 1,800 MHz RF-EMR Exposure Impairs Neurite Outgrowth in Neuronal Cells

**Figure 1:**
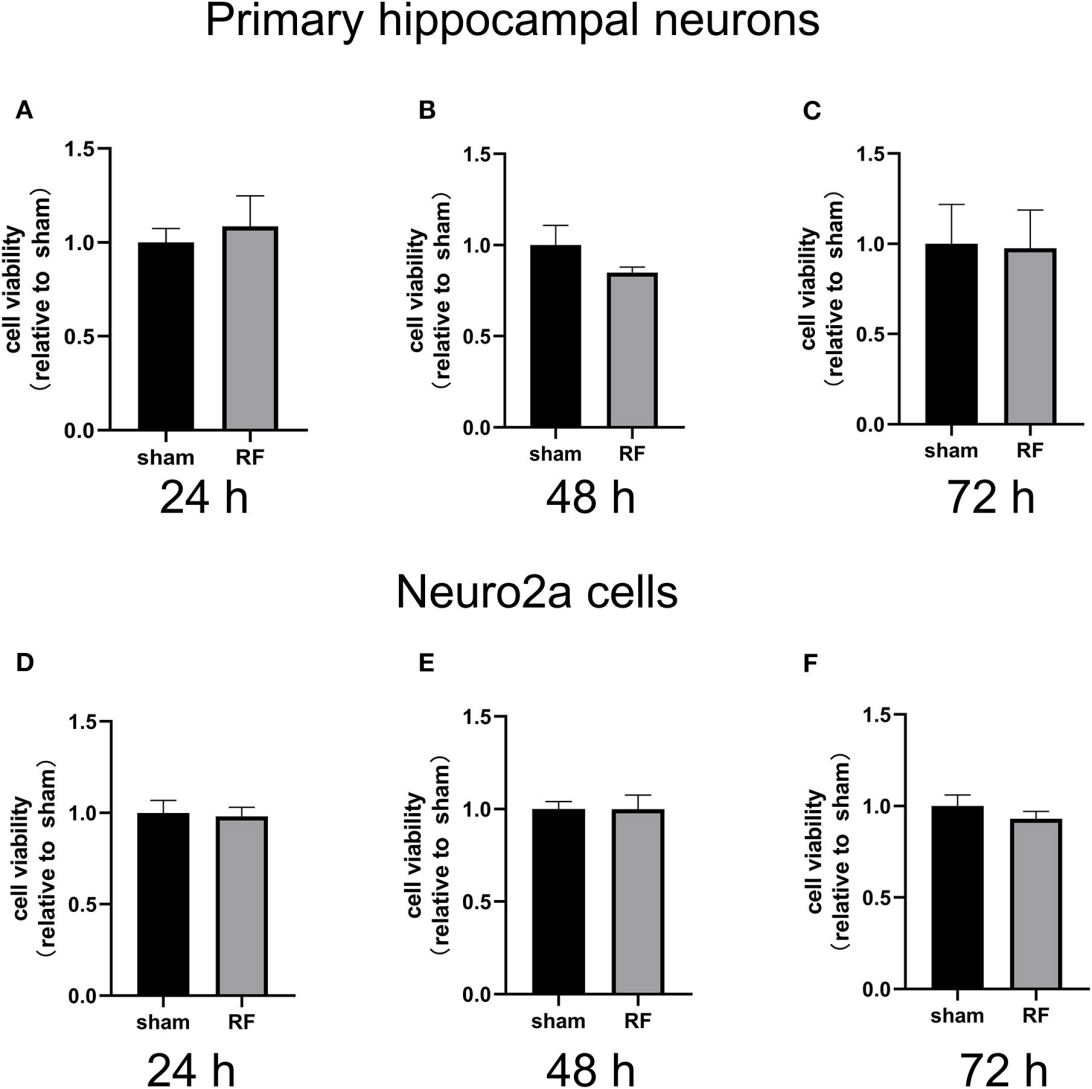
Effects of RF-EMR exposure on neuronal cell viability. Cell viability was determined using a CCK-8 assay in primary mouse hippocampal neurons from C57BL/6 mice **(A–C)** and Neuro2a cells **(D–F)** irradiated by 24-, 48-, and 72-h 1,800 MHz RF-EMR at 4 W/kg. Data are represented as fold changes relative to sham from three independent experiments. The values are presented as the means ± SEM. Student's *t*-test was performed to compare groups.

**Figure 2:**
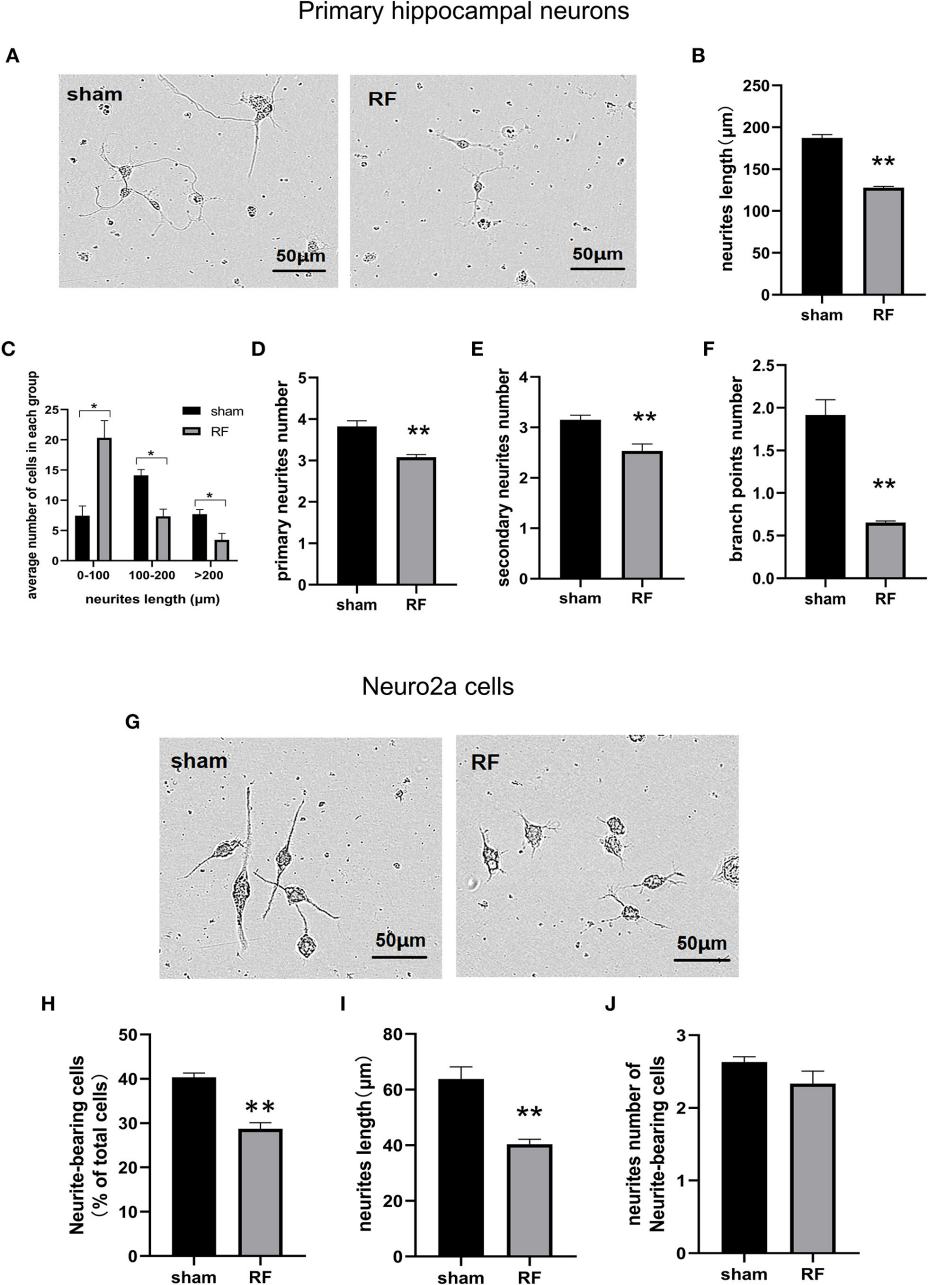
Effects of RF-EMR exposure on neurite outgrowth in neuronal cells. Primary hippocampal neurons **(A–F)** and Neuro2a cells **(G–J)** were exposed to 1,800 MHz RF-EMR at 4 W/kg for 48 h. Morphological parameters were identified according to the description in the methods. Representative images of primary hippocampal neurons **(A)** and Neuro2a cells **(G)** were taken under a (20 ×) Leica microscope, and pictures were analyzed with ImageJ software. **(B)** shows the total neurite length per primary hippocampal neuron, **(C)** shows the number of cells in the groups with a total neurite length per primary hippocampal neuron larger than 100 μm, **(D,E)** shows the primary and secondary neurite numbers per primary hippocampal neuron, **(F)** shows the branch point number per primary hippocampal neuron, **(H)** shows the neurite-bearing cell percentage in Neuro2a cells, **(I)** shows the total neurite length per Neuro2a cell, and **(J)** shows neurite number of neurite-bearing Neuro2a cells after RF-EMR exposure. ^*^P < 0.05, ^**^P < 0.01, Student's *t*-test. Scale bar: 50 μm.

Cell viability was unaffected by 24- to 72-h exposure to 1,800 MHz RF-EMR at 4 W/kg in either primary cultured hippocampal neurons or Neuro2a cells (Figure 1). In primary hippocampal neurons, the total neurite length per cell and the number of primary and secondary branches decreased after 48 h of 1,800 MHz RF-EMR exposure. The average number of branch points of each primary hippocampal neuron decreased after irradiation. The number of neurons with an average total neurite length between 0 and 100 μm increased, while the number of neurons with an average total neurite length between 100 and 200 μm and >200 μm decreased (Figures 2A–F). Similarly, the average total neurite length and neurite-bearing cell percentage of Neuro2a cells stimulated by retinoic acid (RA) decreased after 48 h of 1,800 MHz RF-EMR exposure at 4 W/kg. However, there was no significant difference in the average number of neurites among neurite-bearing Neuro2a cells between the sham and RF-EMR exposure groups (Figures 2G–J). In primary hippocampal neurons, BDNF treatment (positive control) can increase the average neurites length of each neuron by 50% compared with the sham group, and can increase the primary neurites number per neurons. However, PD98059 treatment (negative control) can inhibit the total neurites length of each cell by about 60%, and can decrease the number of primary, secondary neurites and branch points per neuron (Supplementary Figures 1B–F).

### Rap1 Activity Is Decreased After 48-H 1,800 MHz RF-EMR Exposure Without Expression Alteration

**Figure 3:**
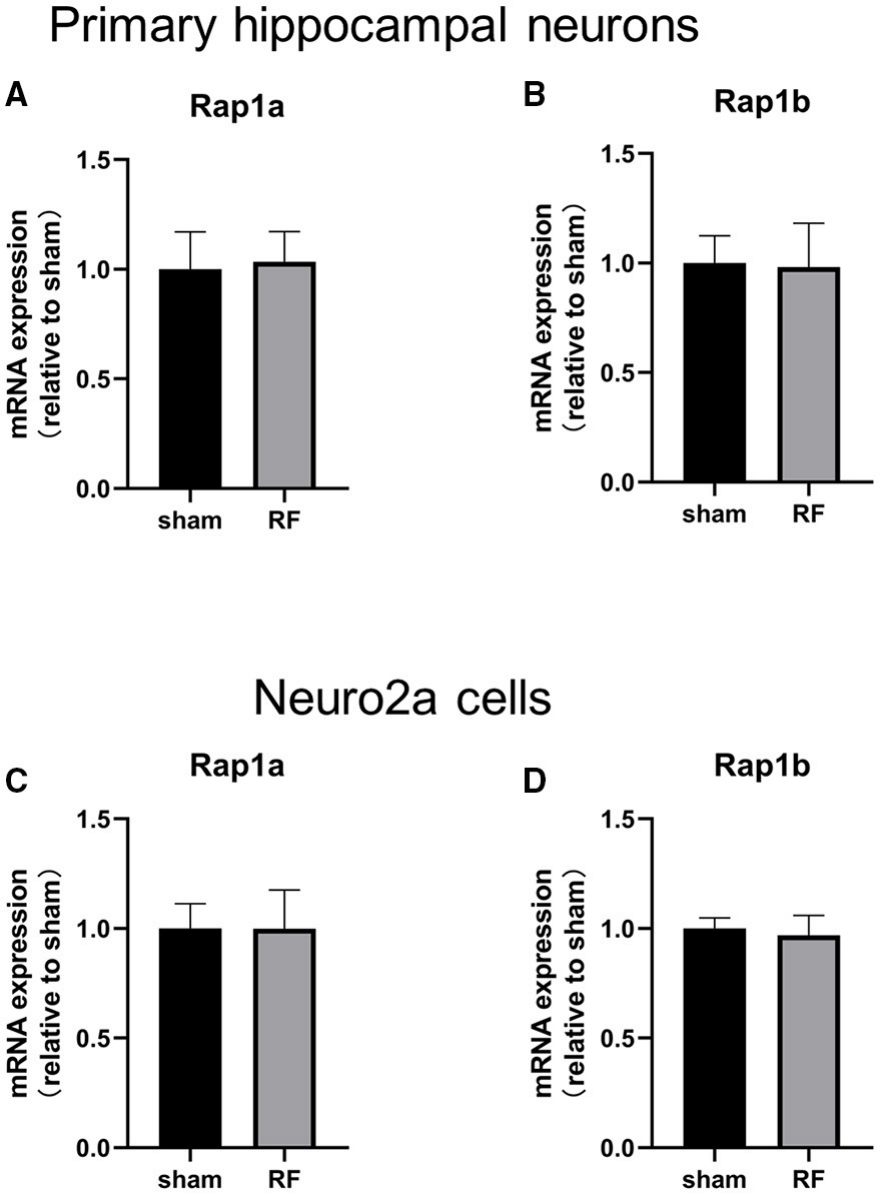
Effects of RF-EMR exposure on the gene expression of Rap1a and Rap1b in neuronal cells. Neuronal cells were exposed to 1,800 MHz RF-EMR for 48 h at 4 W/kg. Gene expression was analyzed using real-time quantitative PCR. The Rap1 gene expression in primary hippocampal neurons **(A,B)** and Neuro2a cells **(C,D)** after RF-EMR exposure.

**Figure 4:**
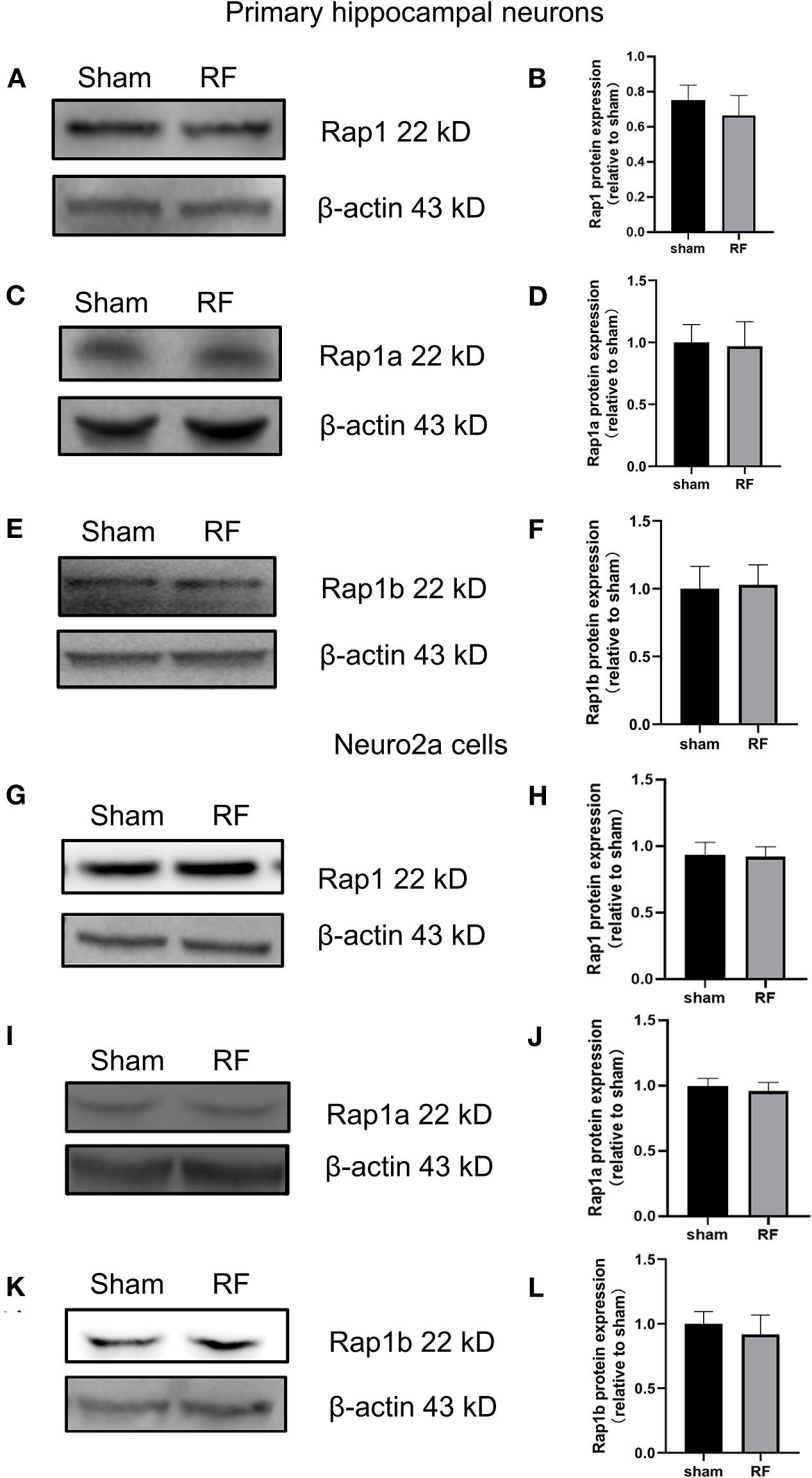
Effects of RF-EMR exposure on the protein expression of Rap1, Rap1a, and Rap1b in neuronal cells. Neuronal cells were exposed to 4 W/kg RF-EMR for 48 h. Protein expression was analyzed using western blot analysis. The protein expression levels of Rap1, Rap1a, and Rap1b in primary hippocampal neurons **(A–F)** and Neuro2a cells **(G–L)** after RF-EMR exposure.

**Figure 5:**
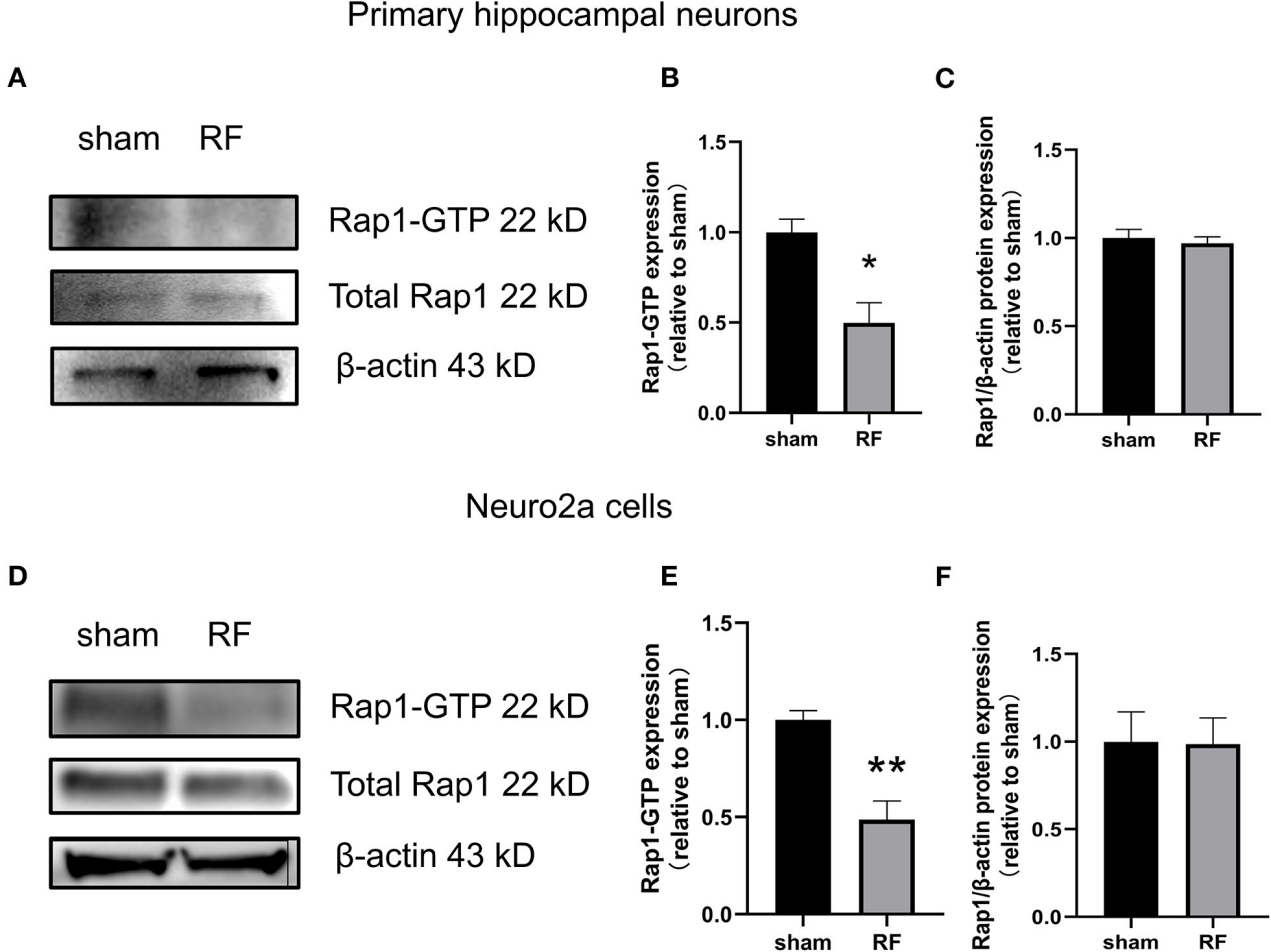
Effects of RF-EMR exposure on Rap1-GTP protein expression in neuronal cells. Neuronal cells were exposed to 4 W/kg RF-EMR for 48 h. The protein expression of Rap1-GTP protein in primary hippocampal neurons **(A–C)** and Neuro2a cells **(D–F)** is shown. Protein expression was analyzed by immunoprecipitation and western blot analysis. ^*^P < 0.05, ^**^P < 0.01, Student's *t*-test.

The gene expression of Rap1a and Rap1b and the protein expression of Rap1, Rap1a, and Rap1b were not significantly altered after 48 h of 1,800 MHz RF-EMR exposure at 4 W/kg in neuronal cells (Figures 3, 4). Immunoprecipitation was used to detect the expression of the active Rap1 form, Rap1-GTP. Rap1-GTP decreased after RF-EMR exposure in both primary cultured hippocampal neurons and Neuro2a cells (Figure 5).

### 48-H 1,800 MHz RF-EMR Exposure Enhances the Protein Expression of Rap1GAP and Decreases the Protein Expression of p-MEK1/2 in Neuronal Cells

**Figure 6:**
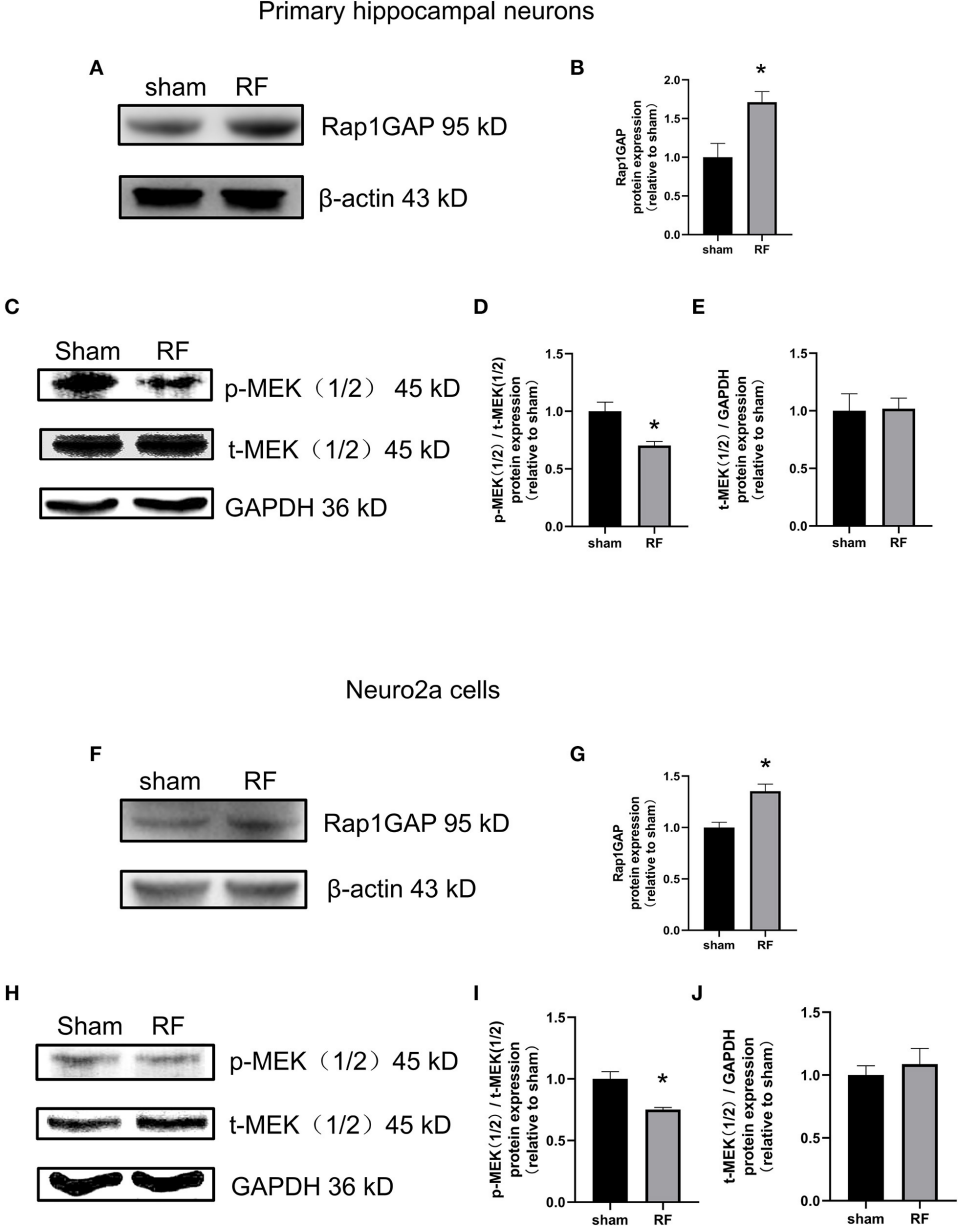
Effects of RF-EMR exposure on the protein expression of Rap1GAP and p-MEK1/2 in neuronal cells. Neuronal cells were exposed to 4 W/kg RF-EMR for 48 h. The protein expression of Rap1GAP in primary hippocampal neurons **(A–E)** and Neuro2a cells **(F–J)** were detected by western blot. **(A,B)** shows protein expression of Rap1GAP in primary hippocampal neurons, and **(F,G)** in Neuro2a cells. The expression levels of MEK1/2 and p-MEK1/2 after RF-EMR exposure for 48 h were detected by western blot analysis in primary hippocampal neurons **(C–E)** and Neuro2a cells **(H–J)**. ^*^P < 0.05, Student's *t*-test.

Rap1-GTP is a molecular switch that can be activated by GEFs and inactivated by GTPase activating proteins (Rap1GAPs). Rap1GAPs are representative molecules inactivating Rap1-GTPase (30). MEK1/2, a downstream molecule of Rap1, is a key molecule in the cascade reaction of the MAPK signaling pathway. When phosphorylated, MEK1/2 can activate downstream MAPK. Rap1-MEK-MAPK signaling can impact neuronal development, plasticity, and survival, especially by regulating neurite outgrowth (31). We found that the protein expression of Rap1GAP was enhanced while that of p-MEK1/2 decreased after 48 h of RF-EMR exposure in neuronal cells (Figure 6).

### Overexpression of Constitutively Active Rap1 Reverses the Neurite Outgrowth Impairment Induced by 48-H RF-EMR Exposure in Neuro2a Cells

**Figure 7:**
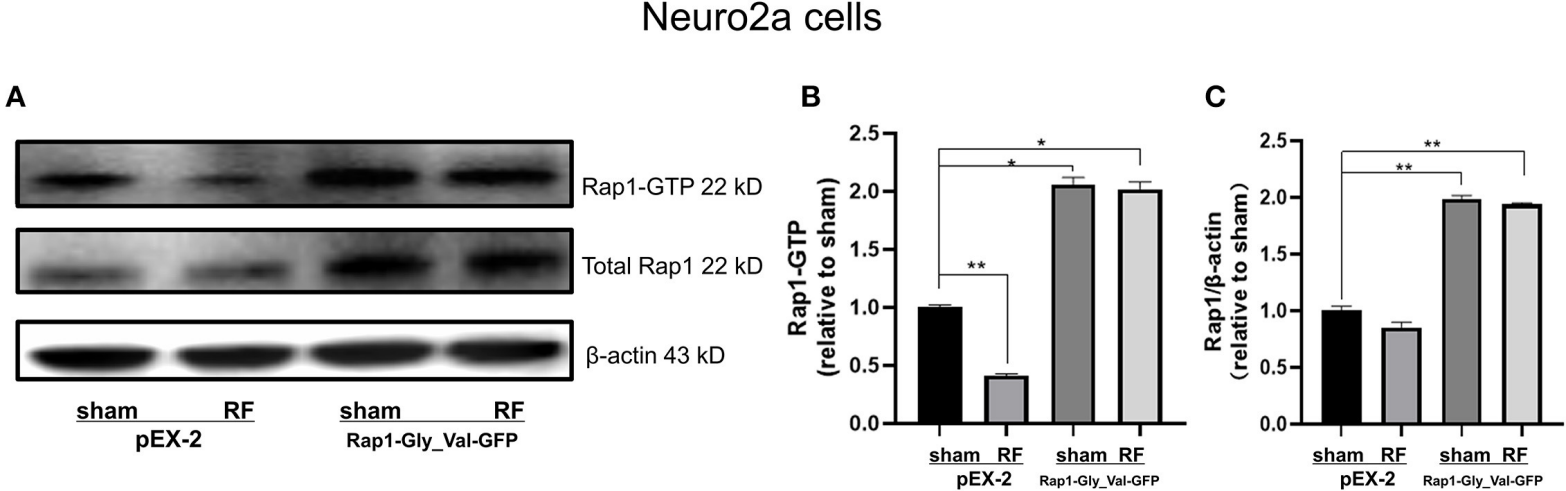
The transfection of constitutively active Rap1 can reverse the decrease in Rap1-GTP induced by 1,800 MHz RF-EMR exposure in Neuro2a cells. Neuro2a cells were transfected by Rap1 constitutively active mutant plasmid (Rap1-Gly_Val-GFP) for 24 h. Transfected Neuro2a cells were exposed to RF-EMR for 48 h at 4 W/kg. The expression of Rap1-GTP was detected by immunoprecipitation. **(A)** is the representative bands of Rap1-GTP, Rap1, and β-actin in each group in Neuro2a cells, **(B)** is the statistical graph made from the data identified according to the gray value and area from the bands. **(C)** is the ratio of protein expression of Rap1 to β-actin in Neuro2a cells. ^*^P < 0.05, ^**^P < 0.01, one-way ANOVA followed by Bonferroni *post hoc* test.

**Figure 8:**
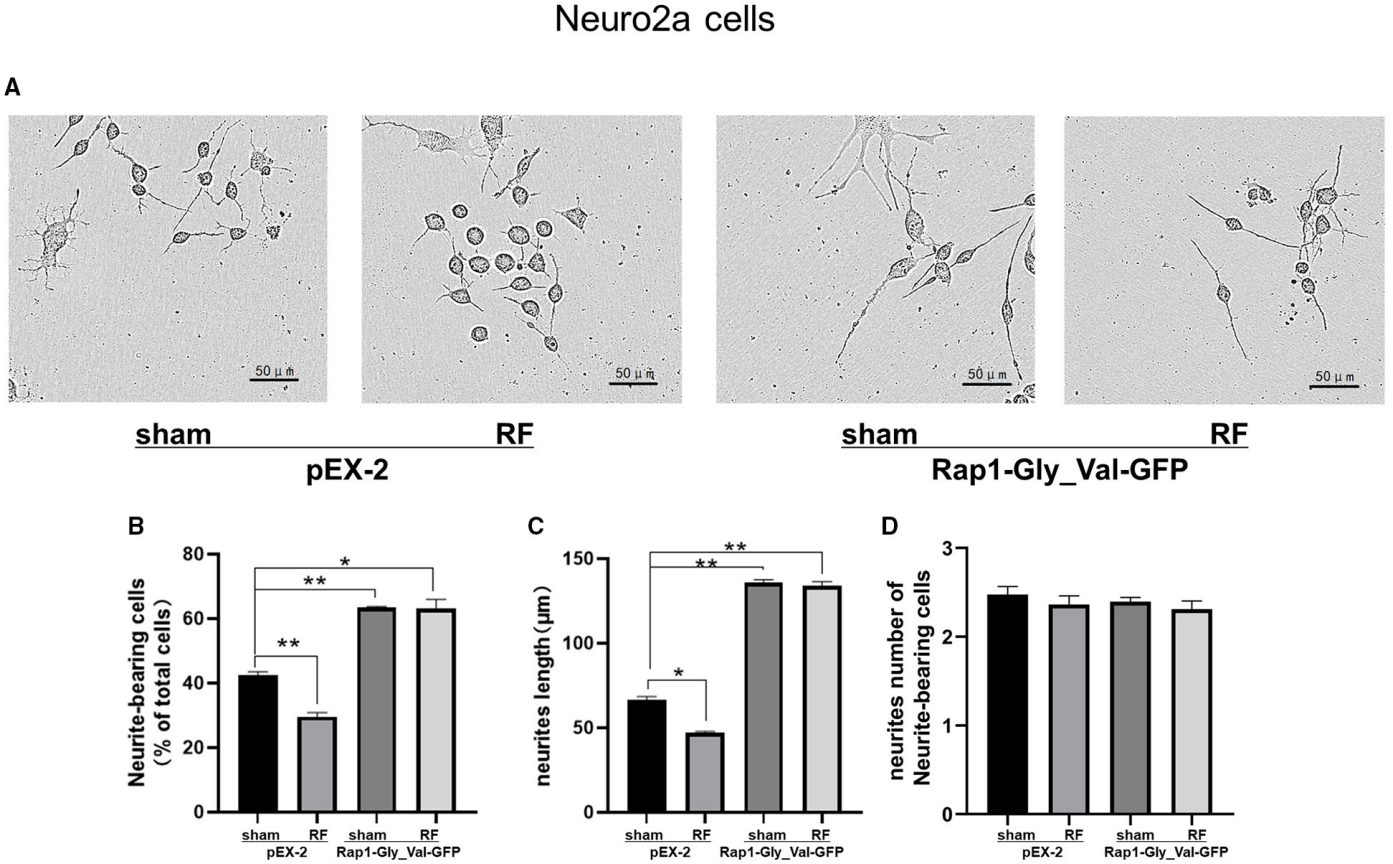
Effects of 1,800 MHz RF-EMR exposure on neurite outgrowth after constitutively active Rap1 transfection in Neuro2a cells. Neuro2a cells were transfected by Rap1 constitutively active mutant plasmid (Rap1-Gly_Val-GFP) for 24 h. The neurite outgrowth of transfected Neuro2a cells induced by RF-EMR exposure for 48 h was assessed by ImageJ software as mentioned before. **(A)** shows representative pictures of transfected Neuro2a cells with or without irradiation. **(B)** shows Neurite-bearing cell ratio, **(C)** shows neurite length per cell, and **(D)** shows neurite number of neurite-bearing cells. ^*^P < 0.05, ^**^P <0.01, one-way ANOVA followed by Bonferroni *post hoc* test.

To further verify the role of Rap1-GTP in the impairment of neurite outgrowth induced by RF-EMR exposure, we overexpressed Rap1-GTP by transfecting a constitutively active mutant Rap1 plasmid (Rap1-Gly_Val-GFP) into Neuro2a cells. Changing the 12th amino acid of the mutant plasmid from glycine to valine constitutively activates Rap1 (32, 33). After transfection of the constitutively active Rap1, its gene and protein expression levels increased in Neuro2a cells (Supplementary Figure 2). After transfection of the mutant plasmid, the expression of Rap1-GTP increased after 48 h of irradiation (Figure 7). Overexpression of Rap1-GTP reversed the disturbance of neurite outgrowth induced by 48 h of RF-EMR exposure (Figure 8).

## Discussion

Neurite outgrowth is an important process of morphological change in neuronal differentiation and development, which is tightly controlled and regulated by several interior and exterior signals. Disturbance of neurite outgrowth will cause defective neural development and neuronal diseases. In our study, we found that 48-h 1,800 MHz RF-EMR exposure interrupted neurite outgrowth in primary cultured hippocampal neurons and Neuro2a cells. Rap1, which plays a critical role in controlling neurite outgrowth and dendrite spine morphology, was inactivated by RF-EMR exposure, while overexpression of constitutively active Rap1-GTP reversed the impairment of neurite outgrowth induced by RF-EMR exposure.

The brain is one of the most sensitive organs to the biological effects of RF-EMR. The results from epidemiological investigations showed that the use of mobile phones can lead to the perceived health impairment in adolescents (34). Another study indicated that mobile phone usage induces anxiety, depression, and stress (35). Animal experiments also showed that RF-EMR exposure causes abnormal neural behavior and impacts dendritic arborization patterns (36). As the brains and skulls of infants and young children are still in developing state, the same electromagnetic background may cause their brains to absorb more electromagnetic energy than adult individuals. The specific absorption by brain tissue in children is 60–125% higher than that in adults (37).

Neurite outgrowth plays a key role in the formation of neural networks during neural development and nerve regeneration after trauma or disease. Neurite outgrowth is regulated by precise signal networks that control processes from sprouting and extending to the formation of axons and dendrites (38, 39). Abnormal neurite outgrowth may cause aberrant polarity, abnormal synaptic plasticity of neuronal cells, and damage to axons and dendrites. Unusual neurodevelopment can induce autism, hyperactivity disorder, dyslexia and so on (40). Deformed neuronal cell morphology may also cause neurodegenerative diseases such as Alzheimer's disease and Parkinson's disease.

In this study, we found that 48-h 1,800 MHz RF-EMR exposure could interfere with neurite outgrowth in hippocampal neurons, including decreasing neurite length and the number of neurite branches. Additionally, neuronal differentiation induced by RA was inhibited by RF-EMR exposure in Neuro2a cells. Kim et al. reported that the total number of dendritic spines was significantly decreased in hippocampal neurons after RF-EMR exposure at 4 W/kg for 4 weeks (9). Our previous studies also showed that 72 h of 1,800 MHz RF-EMR exposure significantly inhibited neurite outgrowth in embryonic neural stem cells (eNSCs) at 4 W/kg, in which the BHLH gene and the EPHA5 signaling pathway were involved (15, 16). The mechanisms underlying the effects of RF-EMR exposure on neurite outgrowth or synaptic plasticity are thought to include changes in calcium ion channels, the myelin sheath, oxidative stress and the glutamate receptor signaling pathway (36, 41, 42). However, additional evidence and further study are required to confirm these hypotheses. This study confirmed that 1,800 MHz RF-EMR exposure affects neurite outgrowth, neurite length, and the number of branch points in each cell, which is consistent with previous studies.

The International Commission on Non-Ionizing Radiation Protection (ICNIRP) sets the limit of local head exposure in the general population at 2 and 10 W/kg for the occupational population (43). The SAR dose of 4 W/kg was usually used to explore the possible biological effects of RF-EMR. Several previous studies have observed the effects of RF-EMR on neuronal cells at SAR value of 4 W/kg, showing the confirmed effect of RF-EMR exposure on dendritic development, cell cycle, etc. (9, 44–46).

Rap1, a member in a small family of monomer GTP-binding proteins, plays a key role in cell proliferation, differentiation, survival, adhesion, and migration (28). Rap1 signaling mediates the role of Semas-Plexins in neuropolarity generation, neurogenesis, axonal growth cone formation, and guidance (47). Rap1-GTP can affect morphological changes in neuronal cells without changing the gene and protein expression of Rap1 (26). In this study, the active form of Rap1, but not its gene and protein expression levels, was decreased after RF-EMR exposure. Altered Rap1-GTP levels were found in the prefrontal cortex of depressed individuals who had committed suicide and in the frontal cortex of patients with schizophrenia and severe depressive disorders (48). Transfection of a constitutively active Rap1 (F64A) plasmid reversed the inhibitory effect of Myelin-associated glycoprotein (MAG) on the neurite growth of dorsal root ganglion (DRG) neurons, possibly by decreasing the expression of Rap1-GTP, limiting neurite outgrowth (25). Rap1 activity is impaired by SynGAP-β, which leads to affected synaptic plasticity and dendrite development in the hippocampus (49). To further verify the role of Rap1-GTP in RF-EMR exposure, we transfected Rap1 mutant plasmid (Rap1-Gly_Val-GFP) to reverse the damage to neurite growth. Compared with indirect Rap1 agonist or inhibitor Rap1, the mutant active Rap1 plasmid can better verify the effect of Rap1 activity. By comparing neurite outgrowth after transfection of the negative control plasmid and constitutively active Rap1 plasmids, we showed that transfection of the constitutively active Rap1 plasmid effectively promoted neurite outgrowth without considering RF-EMR exposure, and transfection of the mutant plasmid also reversed the neurite outgrowth restriction caused by irradiation. Although the neuronal cells transfected with mutant plasmid were irradiated, the neurites still grew well and grew better than the negative control plasmid group without irradiation. The increase in Rap1-GTP could promote neurite outgrowth. This is consistent with the result that transfection of a constitutively active Rap1 plasmid can contribute to neurite complexity (50).

Rap1 links extracellular signals to intracellular signals through second messengers. Rap1 may affect the growth of neurites through a variety of mechanisms. When Rap1 is activated by Ca^2+^ and diacylglycerol-activated guanine nucleotide exchange factors (CalDAG-GEFs), it becomes a downstream molecule of Ca^2+^ signaling pathway. When activated by cAMP-dependent GEF, Rap1 can regulate the concentration of cytoplasmic Ca^2+^ and become its upstream molecule (51). The calcium signaling pathway promotes the growth of neurites, possibly by increasing the activity of calcium binding protein, which reduces the concentration of calcium (52). The interaction between Rap1 and Ca^2+^ also provides a theoretical basis for the regulation of neurite outgrowth of neuronal cells by Rap1. RA-RhoGAP is a direct downstream target of Rap1 in the neurite growth process that possesses a lipid-bound (PH) domain. Inactivity of Rap1 can weaken the GAP activity of Rho, and finally affect nerve axon extension (53). RF-EMR exposure may also reduce the binding of phosphatidylic acid to this PH domain by reducing the activity of Rap1. Rap1-GTP activates PSD-95/discs large/ZO-1 (PDZ)-GEF1 through a positive feedback mechanism (54). So the effect of irradiation on the interaction between Rap1 and PDZ-GEF1 may exist.

Although the above mechanisms regulating Rap1 cannot be ruled out, in this experiment, the expression of Rap1GAP increased after irradiation. Therefore, we consider that the active form of Rap1 may be weakened by Rap1GAP. The active form of Rap1 hydrolyzed by Rap1GAP in non-electromagnetic environment has been reported. Higher level of active Rap1-GTP was found in the brain of Go2 α defective mutant mice, indicating that Go2 α may increase Rap1GAP activity and affect Rap1 activation/inactivation cycle. Go2 α deficient mutant mice developed more branches than those of wild-type mice (27). In striatum medium polyspinous neurons, following activation of the D1 dopamine receptor, Ser441 and Ser499 of Rap1GAP can be phosphorylated by PKA. This inhibits GAP activity and increases Rap1 activity, resulting in better dendritic spine growth (55).

In this study, the expression of phosphorylated form of MEK1/2 decreased after irradiation. We consider that RF-EMR exposure may damage the neurite outgrowth through MEK, the downstream of Rap1 active form. It has been reported that the Rap1-MEK-MAPK signaling pathway promotes the growth of neurites in NG108-15 cells (56). Previous studies have shown that an increase in endogenous β-amyloid peptide leads to an increase in the expression of cAMP response element-related genes and finally promotes synaptic plasticity through the Rap1-MEK signaling pathway in PC12 cells (57). Nerve growth factor also continuously activates ERK through the Rap1-MEK pathway (58). Therefore, RF-EMR exposure may reduce the activity of Rap1 through Rap1GAP and then cause a decrease in p-MEK1/2, resulting in impaired neurite outgrowth.

Despite our findings from this study, more researches need to be done regarding the irradiation-induced decrease in Rap1 activity and how this leads to impaired neurite outgrowth. According to the previous reports, the effect of the transfection of wild-type Rap1 on neurite outgrowth is very limited, so in this study we applied a constitutively active Rap1 plasmid to verify the role of Rap1-GTP in neurite outgrowth impairment induced by RF-EMR exposure (59).

Rap1 is activated by a specific GEF, the role of which in RF-EMR exposure-impaired neurite outgrowth needs to be investigated by further researches, as does the specific regulation of Rap1GAP after irradiation. As Rap1 activity is influenced by RF-EMR exposure and the constitutively active Rap1 can rescue neurite outgrowth impairment induced by RF-EMR, Rap1 may be a potential new candidate for interfering the disruption of developing brain by RF-EMR exposure.

## Conclusion

We found that Rap1 is involved in the disturbance of neurite outgrowth induced by 1,800 MHz RF-EMR exposure in neuronal cells. Due to the developmental sensitivity of infants and adolescents, the neuronal impairment induced by 1,800 MHz RF-EMR exposure can interrupt programmatic neural development and cause abnormal neuronal behavior and diseases. Rap1 activity and related signaling pathways may be critical targets in the neuronal effects induced by RF-EMR exposure. The influence of RF-EMR exposure on the developing brain requires greater attention.

## Materials and Methods

### Cell Culture

A mouse neuroblastoma cell line (Neuro2a) (Chunmai, China) was maintained at 37.0 ± 0.2°C and 5% CO_2_. Neuro2a cells were cultured in high-glucose Dulbecco's modified Eagle's medium (DMEM; Sigma, USA) with 10% fetal bovine serum (FBS; Biological Industries, ISR) and 50 mg/ml penicillin-streptomycin (Sigma, USA). Cells were subcultured every 2 days. Neuro2a cells were seeded in 35 mm plastic dishes (Thermo Scientific, USA) for 12 h before exposure to RF-EMR exposure. Neuro2a cells induced by retinoic acid were used. This model has been proved to be efficient in several studies (22, 60). The culture medium was changed to differentiation medium immediately before irradiation. The differentiation medium was composed of 99% DMEM, 1% FBS, and 10 μM retinoic acid (Sigma, USA). FBS and retinoic acid concentration were slightly regulated to obtain optimized differentiation effects according to different cell density. The seeding densities of Neuro2a cells were 3 ×10^4^ cells/35 mm dish for cell viability assay, 2 ×10^5^ cells/35 mm dish for neurite outgrowth analysis, 5 ×10^5^ cells/35 mm dish for RNA, protein extraction and transfection.

Primary hippocampal neurons were prepared from newborn C57/BL mice within 1–3 days of birth. Dissociated neurons were plated in 35-mm dishes with the seeding medium containing 79% DMEM/F12 (Sigma, USA), 20% fetal bovine serum (FBS; Biological Industries, ISR), and 1% penicillin-streptomycin (50 mg/ml) (Sigma, USA). After 12 h of seeding, the culture medium was changed to the maintenance medium consisted of 95% Neurobasal-A Medium (Gibco, USA), 2% B-27 plus supplement (Gibco, USA), 1% culture one supplement (Gibco, USA), 1% Glutamax (Gibco, USA), and 1% penicillin-streptomycin (Gibco, USA). The seeding densities of primary hippocampal neurons were 2 ×10^5^ cells/35 mm dish for neurite outgrowth analysis, 10^6^ cells/35 mm dish for cell viability, RNA and protein extraction.

The animal study was reviewed and approved by Laboratory Animal Welfare and Ethics Committee of Third Military Medical University (AMUWEC20210443).

### Radiofrequency Electromagnetic Irradiation (RF-EMR) at 1,800 MHz

Primary mouse hippocampal neurons and Neuro2a cells were irradiated in an sXc-1800 exposure system (IT'IS Foundation, Zurich, Switzerland) using the irradiation method introduced in previous studies (15, 16). RF-EMR exposure system is mainly composed of the following four parts: narrowband amplifier, arbitrary function generator, RF generator, and two waveguide. Each chamber is equipped with a plastic holder hosting 6 dishes arranged in two stacks. A total of 6 dishes were exposed, 6 dishes in the other chamber served as the sham group. The metal housing of the two chambers ensures that radiation exists only indoors and does not interfere with the outdoors. The operation of fans in chambers can avoid the thermal effect caused by irradiation (61). The sensors and fans in the exposure system were connected to a computer that monitored the SAR value during exposure and maintained a constant temperature and environment for the waveguides (37°C, 5% CO_2_, 95% atmospheric air). 35mm dishes were placed in the H-field maxima and exposed to a polarized E-field (an electric field perpendicular to the H-field). To perform double-blind experiments, the computer randomly determines which of the two waveguides was exposed in each trial. Experiments were also conducted in blind modality where the researchers who performed biological assays did not know which of the two chambers was active, and which acted as sham. RF-EMR exposure was delivered using the GSM talk signal mode at an exposure interval of 5 min irradiation field on and 10 min irradiation field off and an SAR value of 4 W/kg.

### Cell Viability Assay

Cell viability was evaluated by a CCK-8 (Dojindo, Japan) assay following the manufacturer's instructions. Briefly, after 24-, 48-, and 72- h irradiation, 10% CCK-8 solution was added to the medium and incubated at 37°C for 3 h. The absorbance at 450 nm was read by a microplate reader (Tecan, Austria).

### Neurite Outgrowth Analysis

Neurite outgrowth analysis was performed as previously described (15). Briefly, primary hippocampal neurons and Neuro2a cells were cultured in 35-mm dishes, exposed to RF-EMR for 48 h. Neurite outgrowth of primary hippocampal neurons (DIV 2) and Neuro2a cells was observed with a Leica microscope (20 ×). Forty nanogram per milliliter BDNF (MCE, USA) was used as positive control and 50 μM MEK1/2 inhibitor PD98059 (SIGMA, USA) was used as negative control (62, 63). BDNF and PD98059 were added into the maintenance medium and incubated with the neurons for 48 h, respectively. ImageJ software was used to evaluate neurite outgrowth. Thirty cells per group were identified among the primary hippocampal neurons, and 100 cells per group were counted among the Neuro2a cells. The total length of the neurites in each neuronal cell was measured from the cell center to the end of the neurite. The primary neurite was identified as the directly separated process from the cell body, the secondary neurite was the process separated from the primary neurite, and the remaining branches were the branch points. Neuro2a cells with arbitrary neurite lengths greater than the cell body diameter were recognized as neurite-bearing cells. Neurites whose length was greater than the cell body diameter were counted as neurites of neurite-bearing cells.

### Real-Time Quantitative PCR

TRIzol reagent (Takara, JPN) was used to extract total RNA from neuronal cells after exposure to RF-EMR for 48 h. cDNA was obtained by RT-qPCR performed on a CFX96TM real-time system (Bio-Rad, USA) using TB green (Takara, JPN). The Rap1a and Rap1b gene-specific primers used are shown in Supplementary Table 1. The gene expression fold change was calculated and normalized to the gene expression of endogenous β-actin. Then, the relative gene expression level was calculated with reference to the sham.

### Western Blot Analysis

After RF-EMR exposure for 48 h, protein samples were obtained using RIPA buffer (Beyotime, China) containing protease and phosphatase inhibitors (Roche, USA). Twelve percent SDS-PAGE (Genscript, USA), PVDF membranes (Bio-Rad, USA), Quick Block Blocking Buffer (Beyotime, China), TBST solution (Thermo Fisher, USA), and primary and secondary antibodies (details in Supplementary Table 2) were used for western blot analysis. The PVDF membrane was scanned on a chemiDoc XRS+ machine (Bio-Rad, USA) by using ECL luminescent solution (Thermo Fisher, USA).

### Rap1 Activity Assay

The activity of Rap1 was measured by immunoprecipitation after RF-EMR exposure for 48 h (64). The irradiated primary hippocampal neurons and Neuro2a cells were lysed with IP lysate (Beyotime, China) on ice for 15 min, rotated and then centrifuged at 15,000 g for 30 min at 4°C to remove the precipitate. The protein concentration of the supernatant was measured by the BCA method (Beyotime, China). Three hundred micrograms of protein supernatant from each group was taken equally. IgG antibody (Beyotime, China) and 1 μg active Rap1 antibody was added to the sham group and irradiation group and rotated overnight. The next day, protein A/G agarose beads (Beyotime, China) were added to bind the FC segment of active Rap1 antibody to precipitate, and the non-specifically bound antigen was washed away with PBS (Beyotime, China). After adding loading buffer (Beyotime, China), IP samples were heated to denature and subjected to western blot analysis. In the above steps, the IgG group represents the background gray value, and the irradiated group and the control group represent the natural active form of Rap1.

### Transfection of Constitutively Active Rap1 Mutant Plasmid

Glycine at the 12th position of the Rap1 gene was replaced with valine, and GFP fluorescence label was added. The steps for synthesizing the mutant plasmid were introduced in a previous article (31). The mutant plasmid (GenePharma, China) was transfected with the pEX-2 vector. Lipofectamine 3000 transfection reagent (Thermo Scientific, USA) and Opti-MEM medium (Invitrogen, USA) were used to help the mutant plasmids transfecting into cells and stabilize the expression in Neuro2a cells. In the sham group, negative control plasmid carrying the pEX-2 vector was transfected into the cells in the same way. After seeding for 24 h, the transfection reagent and mutant plasmid were added and incubated. After transfection for 24 h, retinoic acid was used for differentiation and irradiation for 48 h as depicted before.

### Statistical Analysis

Statistical analysis was performed using GRAPH Prism 8.0 software. All data were collected from at least three independent duplicate experiments and are presented as the mean ± standard error of the mean (SEM). Student's *t*-tests, one-way ANOVA followed by the Brown–Forsythe test and Welch ANOVA were used to determine significance. *P* < 0.05 was considered statistically significant.

## Data Availability Statement

The original contributions presented in the study are included in the article/Supplementary Material, further inquiries can be directed to the corresponding author.

## Ethics Statement

The animal study was reviewed and approved by Laboratory Animal Welfare and Ethics Committee of Third Military Medical University (AMUWEC20210443).

## Author Contributions

LZ and ZY designed the experiment and conceived the manuscript. YaL, PD, CZ, and ZH did the experiments. YaL, CC, and HP analyzed the data. YaL and LZ wrote the draft of the manuscript. MH, PG, YZ, and QM reviewed the manuscript. LZ and ZY critically revised the manuscript. All authors listed have made a substantial, direct and intellectual contribution to the work, and approved it for publication.

## Funding

This research was supported by the National Natural Science Foundation of China (Grant Nos. 31670854 and 31770906).

## Conflict of Interest

The authors declare that the research was conducted in the absence of any commercial or financial relationships that could be construed as a potential conflict of interest.

## Publisher's Note

All claims expressed in this article are solely those of the authors and do not necessarily represent those of their affiliated organizations, or those of the publisher, the editors and the reviewers. Any product that may be evaluated in this article, or claim that may be made by its manufacturer, is not guaranteed or endorsed by the publisher.

## References

[1] Pearce J. Limiting liability with positioning to minimize negative health effects of cellular phone towers. Environ Res. (2020) 181:108845. 10.1016/j.envres.2019.108845 31791710

[2] Szilágyi Z Németh Z Bakos J Necz P Sáfár A Kubinyi G. Evaluation of inflammation by cytokine production following combined exposure to ultraviolet and radiofrequency radiation of mobile phones on 3D reconstructed human skin *in vitro*. Int J Environ Res Public Health. (2020) 17:4401. 10.3390/ijerph17124401 32575398 PMC7344923

[3] Lee J Jang S Ju Y Kim W Lee H Park E. Relationship between mobile phone addiction and the incidence of poor and short sleep among korean adolescents: a longitudinal study of the Korean Children & Youth Panel Survey. J Korean Med Sci. (2017) 32:1166–72. 10.3346/jkms.2017.32.7.1166 28581275 PMC5461322

[4] Ishihara T Yamazaki K Araki A Teraoka Y Tamura N Hikage T. Exposure to radiofrequency electromagnetic field in the high-frequency band and cognitive function in children and adolescents: a literature review. Int J Environ Res Public Health. (2020) 17:9179. 10.3390/ijerph17249179 33302600 PMC7764655

[5] Hardell L. Effects of mobile phones on children's and adolescents' health: a commentary. Child Dev. (2018) 89:137–40. 10.1111/cdev.12831 28504422

[6] Miller A Sears M Morgan L Davis D Hardell L Oremus M. Risks to health and well-being from radio-frequency radiation emitted by cell phones and other wireless devices. Front Public Health. (2019) 7:223. 10.3389/fpubh.2019.00223 31457001 PMC6701402

[7] Zheng F Gao P He M Li M Tan J Chen D. Association between mobile phone use and self-reported well-being in children: a questionnaire-based cross-sectional study in Chongqing, China. BMJ Open. (2015) 5:e007302. 10.1136/bmjopen-2014-007302 25967996 PMC4431134

[8] Zheng F Gao P He M Li M Wang C Zeng Q. Association between mobile phone use and inattention in 7102 Chinese adolescents: a population-based cross-sectional study. BMC Public Health. (2014) 14:1022. 10.1186/1471-2458-14-1022 25273315 PMC4190308

[9] Kim J Chung K Hwang Y Park H Kim H Kim H. Exposure to RF-EMF alters postsynaptic structure and hinders neurite outgrowth in developing hippocampal neurons of early postnatal mice. Int J Molecular Sci. (2021) 22:5340. 10.3390/ijms22105340 34069478 PMC8159076

[10] O'Connor R Madison S Leveque P Roderick H Bootman M. Exposure to GSM RF fields does not affect calcium homeostasis in human endothelial cells, rat pheocromocytoma cells or rat hippocampal neurons. PLoS ONE. (2010) 5:e11828. 10.1371/journal.pone.0011828 20676401 PMC2910734

[11] Liu M Wen J Fan Y. Potential protection of green tea polyphenols against 1800 MHz electromagnetic radiation-induced injury on rat cortical neurons. Neurotox Res. (2011) 20:270–6. 10.1007/s12640-011-9240-4 21293955

[12] El Khoueiry C Moretti D Renom R Camera F Orlacchio R Garenne A. Decreased spontaneous electrical activity in neuronal networks exposed to radiofrequency 1,800 MHz signals. J Neurophysiol. (2018) 120:2719–29. 10.1152/jn.00589.2017 30133383

[13] Su L Yimaer A Xu Z Chen G. Effects of 1800 MHz RF-EMR exposure on DNA damage and cellular functions in primary cultured neurogenic cells. Int J Radiat Biol. (2018) 94:295–305. 10.1080/09553002.2018.1432913 29368975

[14] Ning W Xu S Chiang H Xu Z Zhou S Yang W. Effects of GSM 1800 MHz on dendritic development of cultured hippocampal neurons. Acta Pharmacol Sin. (2007) 28:1873–80. 10.1111/j.1745-7254.2007.00668.x 18031599

[15] Chen C Ma Q Liu C Deng P Zhu G Zhang L. Exposure to 1800 MHz radiofrequency radiation impairs neurite outgrowth of embryonic neural stem cells. Sci Rep. (2014) 4:5103. 10.1038/srep05103 24869783 PMC4037711

[16] Chen C Ma Q Deng P Lin M Gao P He M. 1800 MHz radiofrequency electromagnetic field impairs neurite outgrowth through inhibiting EPHA5 signal. Front Cell Dev Biol. (2021) 9:657623. 10.3389/fcell.2021.657623 33912567 PMC8075058

[17] Lekhraj R Cynamon D DeLuca S Taub E Pilla A Casper D. Pulsed electromagnetic fields potentiate neurite outgrowth in the dopaminergic MN9D cell line. J Neurosci Res. (2014) 92:761–71. 10.1002/jnr.23361 24523147

[18] Inoue S Motoda H Koike Y Kawamura K Hiragami F Kano Y. Microwave irradiation induces neurite outgrowth in PC12m3 cells via the p38 mitogen-activated protein kinase pathway. Neurosci Lett. (2008) 432:35–9. 10.1016/j.neulet.2007.12.002 18166272

[19] Wang K Lu J Xing Z Zhao Q Hu L Xue L. Effect of 18 GHz radiofrequency electromagnetic radiation on novel object associative recognition memory in mice. Sci Rep. (2017) 7:44521. 10.1038/srep44521 28303965 PMC5355939

[20] Li W Ma K Jiang X Yang R Lu P Nie B. Molecular mechanism of panaxydol on promoting axonal growth in PC12 cells. Neural Regen Res. (2018) 13:1927–36. 10.4103/1673-5374.239439 30233066 PMC6183029

[21] Jaśkiewicz A Pajak B Orzechowski A. The many faces of Rap1 GTPase. Int J Mol Sci. (2018) 19:2848. 10.3390/ijms19102848 30241315 PMC6212855

[22] Huang M Liang C Li S Zhang J Guo D Zhao B. DOCK4 two autism/dyslexia linked variations of disrupt the gene function on Rac1/Rap1 activation, neurite outgrowth, and synapse development. Front Cell Neurosci. (2019) 13:577. 10.3389/fncel.2019.00577 32009906 PMC6974517

[23] Yang D Roh S Jeong S. The axon guidance function of Rap1 small GTPase is independent of PlexA RasGAP activity in Drosophila. Dev Biol. (2016) 418:258–67. 10.1016/j.ydbio.2016.08.026 27565025

[24] Shah B Lutter D Tsytsyura Y Glyvuk N Sakakibara A Klingauf J. Rap1 GTPases are master regulators of neural cell polarity in the developing neocortex. Cerebr Cortex. (2017) 27:1253–69. 10.1093/cercor/bhv341 26733533

[25] Nikulina E Gkioka V Siddiq M Mellado W Hilaire M Cain C. Myelin-associated glycoprotein inhibits neurite outgrowth through inactivation of the small GTPase Rap1. FEBS Lett. (2020) 594:1389–402. 10.1002/1873-3468.13740 31985825 PMC7217748

[26] Yin N Hong X Han Y Duan Y Zhang Y Chen Z. Cortex Mori Radicis Extract induces neurite outgrowth in PC12 cells activating ERK signal pathway via inhibiting Ca(2+) influx. Int J Clin Exp Med. (2015) 8:5022–32. 10.3892/mmr.2019.10839 26131075 PMC4483855

[27] Baron J Blex C Rohrbeck A Rachakonda S Birnbaumer L Ahnert-Hilger G. The α-subunit of the trimeric GTPase Go2 regulates axonal growth. J Neurochem. (2013) 124:782–94. 10.1111/jnc.12123 23373526 PMC3593993

[28] Guimond M Wallinder C Alterman M Hallberg A Gallo-Payet N. Comparative functional properties of two structurally similar selective nonpeptide drug-like ligands for the angiotensin II type-2 (AT(2)) receptor. Effects on neurite outgrowth in NG108-15 cells. Euro J Pharmacol. (2013) 699:160–71. 10.1016/j.ejphar.2012.11.032 23211679

[29] Guimond M Roberge C Gallo-Payet N. Fyn is involved in angiotensin II type 2 receptor-induced neurite outgrowth, but not in p42/p44mapk in NG108-15 cells. Mol Cell Neurosci. (2010) 45:201–12. 10.1016/j.mcn.2010.06.011 20600928

[30] Spilker C Kreutz M. RapGAPs in brain: multipurpose players in neuronal Rap signalling. Eur J Neurosci. (2010) 32:1–9. 10.1111/j.1460-9568.2010.07273.x 20576033

[31] Lin Y Yamahashi Y Kuroda K Faruk M Zhang X Yamada K. Accumbal D2R-medium spiny neurons regulate aversive behaviors through PKA-Rap1 pathway. Neurochem Int. (2021) 143:104935. 10.1016/j.neuint.2020.104935 33301817

[32] Fu Z Lee S Simonetta A Hansen J Sheng M Pak D. Differential roles of Rap1 and Rap2 small GTPases in neurite retraction and synapse elimination in hippocampal spiny neurons. J Neurochem. (2007) 100:118–31. 10.1111/j.1471-4159.2006.04195.x 17227435 PMC12125706

[33] Kitayama H Matsuzaki T Ikawa Y Noda M. Genetic analysis of the Kirsten-ras-revertant 1 gene: potentiation of its tumor suppressor activity by specific point mutations. Proc Natl Acad Sci USA. (1990) 87:4284–8. 10.1073/pnas.87.11.4284 2112251 PMC54093

[34] Cho Y Lim H Jang H Kim K Choi J Shin C. A follow-up study of the association between mobile phone use and symptoms of ill health. Environ Health Toxicol. (2016) 32:e2017001. 10.5620/eht.e2017001 28111420 PMC5365277

[35] Gao T Li J Zhang H Gao J Kong Y Hu Y. The influence of alexithymia on mobile phone addiction: the role of depression, anxiety and stress. J Affect Disord. (2018) 225:761–6. 10.1016/j.jad.2017.08.020 28926906

[36] Narayanan S Mohapatra N John PKN Kumar R Nayak S. Radiofrequency electromagnetic radiation exposure effects on amygdala morphology, place preference behavior and brain caspase-3 activity in rats. Environ Toxicol Pharmacol. (2018) 58:220–9. 10.1016/j.etap.2018.01.009 29413766

[37] Lee A Park J Hong S Taki M Wake K Wiart J. Brain SAR of average male Korean child to adult models for mobile phone exposure assessment. Phys Med Biol. (2019) 64:045004. 10.1088/1361-6560/aafcdc 30719982

[38] Kim H Triplet E Radulovic M Bouchal S Kleppe L Simon W. The thrombin receptor modulates astroglia-neuron trophic coupling and neural repair after spinal cord injury. Glia. (2021). 10.1002/glia.24012 PMC8672305 33887067

[39] Damenti M Coceano G Pennacchietti F Bodén A Testa I. STED and parallelized RESOLFT optical nanoscopy of the tubular endoplasmic reticulum and its mitochondrial contacts in neuronal cells. Neurobiol Dis. (2021) 155:105361. 10.1016/j.nbd.2021.105361 33857635

[40] Jeanne M Demory H Moutal A Vuillaume M Blesson S Thépault R. Missense variants in DPYSL5 cause a neurodevelopmental disorder with corpus callosum agenesis and cerebellar abnormalities. Am J Hum Genet. (2021) 108:951–61. 10.1016/j.ajhg.2021.04.004 33894126 PMC8206156

[41] Kim J Lee J Kim H Kim K Kim H. Possible effects of radiofrequency electromagnetic field exposure on central nerve system. Biomol Ther. (2019) 27:265–75. 10.4062/biomolther.2018.152 30481957 PMC6513191

[42] Gökçek-Saraç Ç Er H Kencebay Manas C Kantar Gok D Özen S Derin N. Effects of acute and chronic exposure to both 900 MHz and 2100 MHz electromagnetic radiation on glutamate receptor signal pathway. Int J Radiat Biol. (2017) 93:980–9. 10.1080/09553002.2017.1337279 28565929

[43] Guidelines for Limiting Exposure to Electromagnetic Fields (100 kHz to 300 GHz). Health phys. (2020) 118:483–524. 10.1097/HP.0000000000001210 32167495

[44] Kim J Jeon S Choi H Lee J Bae J Kim N. Exposure to long-term evolution radiofrequency electromagnetic fields decreases neuroblastoma cell proliferation via Akt/mTOR-mediated cellular senescence. J Toxicol Environ Health A. (2021) 84:1–12. 10.1080/15287394.2021.1944944 34196262

[45] Zuo W Hu Y Yang Y Zhao X Zhang Y Kong W. Sensitivity of spiral ganglion neurons to damage caused by mobile phone electromagnetic radiation will increase in lipopolysaccharide-induced inflammation *in vitro* model. J Neuroinflammation. (2015) 12:105. 10.1186/s12974-015-0300-1 26022358 PMC4458026

[46] Kim J Huh Y Kim H. Trafficking of synaptic vesicles is changed at the hypothalamus by exposure to an 835 MHz radiofrequency electromagnetic field. Gen Physiol Biophys. (2019) 38:379–88. 10.4149/gpb_2019020 31411574

[47] Wang N Dhumale P Chiang J Püschel A. The Sema3A receptor Plexin-A1 suppresses supernumerary axons through Rap1 GTPases. Sci Rep. (2018) 8:15647. 10.1038/s41598-018-34092-5 30353093 PMC6199275

[48] Kermath B Vanderplow A Bjornson K Seablom E Novak A Bernhardt C. The Rap1 small GTPase is a critical mediator of the effects of stress on prefrontal cortical dysfunction. Mol Psychiatry. (2020) 26:3223–39. 10.1038/s41380-020-0835-0 32651478 PMC12359851

[49] Araki Y Hong I Gamache T Ju S Collado-Torres L Shin J. SynGAP isoforms differentially regulate synaptic plasticity and dendritic development. ELife. (2020) 9:E56273. 10.7554/eLife.56273 32579114 PMC7314543

[50] Chen Y Wang P Ghosh A. Regulation of cortical dendrite development by Rap1 signaling. Mol Cell Neurosci. (2005) 28:215–28. 10.1016/j.mcn.2004.08.012 15691704

[51] Kosuru R Chrzanowska M. Integration of Rap1 and calcium signal. Int J Mol Sci. (2020) 21:1616. 10.3390/ijms21051616 32120817 PMC7084553

[52] Kusakabe M Hasegawa Y. Nimodipine promotes neurite outgrowth and protects against neurotoxicity in PC12 cells. Iran J Basic Med Sci. (2021) 24:51–7. 10.22038/ijbms.2020.48567.11152 33643570 PMC7894639

[53] Kurooka T Yamamoto Y Takai Y Sakisaka T. Dual regulation of RA-RhoGAP activity by phosphatidic acid and Rap1 during neurite outgrowth. J Biol Chem. (2011) 286:6832–43. 10.1074/jbc.M110.183772 21169361 PMC3057846

[54] Hisata S Sakisaka T Baba T Yamada T Aoki K Matsuda M. Rap1-PDZ-GEF1 interacts with a neurotrophin receptor at late endosomes, leading to sustained activation of Rap1 and ERK and neurite outgrowth. J Cell Biol. (2007) 178:843–60. 10.1083/jcb.200610073 17724123 PMC2064548

[55] McAvoy T Zhou M Greengard P Nairn A. Phosphorylation of Rap1GAP, a striatally enriched protein, by protein kinase A controls Rap1 activity and dendritic spine morphology. Proc Natl Acad Sci USA. (2009) 106:3531–6. 10.1073/pnas.0813263106 19218462 PMC2651273

[56] Beaudry H Gendron L Guimond M Payet M Gallo-Payet N. Involvement of protein kinase C alpha (PKC alpha) in the early action of angiotensin II type 2 (AT2) effects on neurite outgrowth in NG108-15 cells: AT2-receptor inhibits PKC alpha and p21ras activity. Endocrinology. (2006) 147:4263–72. 10.1210/en.2006-0411 16740968

[57] Echeverria V Ducatenzeiler A Chen C Cuello A. Endogenous beta-amyloid peptide synthesis modulates cAMP response element-regulated gene expression in PC12 cells. Neuroscience. (2005) 135:1193–202. 10.1016/j.neuroscience.2005.06.057 16181736

[58] York R Yao H Dillon T Ellig C Eckert S McCleskey E. Rap1 mediates sustained MAP kinase activation induced by nerve growth factor. Nature. (1998) 392:622–6. 10.1038/33451 9560161

[59] Jeon C Moon M Kim J Kim H Kim J Li Y. Control of neurite outgrowth by RhoA inactivation. J Neurochem. (2012) 120:684–98. 10.1111/j.1471-4159.2011.07564.x 22035369

[60] Tremblay R Sikorska M Sandhu J Lanthier P Ribecco-Lutkiewicz M Bani-Yaghoub M. Differentiation of mouse Neuro 2A cells into dopamine neurons. J Neurosci Methods. (2010) 186:60–7. PubMed PMID: 1990349 10.1016/j.jneumeth.2009.11.004 19903493

[61] Schuderer J Samaras T Oesch W Spat D Kuster N. High peak SAR exposure unit with tight exposure and environmental control for *in vitro* experiments at 1800 MHz. IEEE Trans Microw Theory Tech. (2004) 52:2057–66. 10.1109/TMTT.2004.83200927295638

[62] Labelle C Leclerc N. Exogenous BDNF, NT-3 and NT-4 differentially regulate neurite outgrowth in cultured hippocampal neurons. Brain Res Dev Brain Res. (2000) 123:1–11. 10.1016/S0165-3806(00)00069-9 11020545

[63] Veeranna Amin N Ahn N Jaffe H Winters C Grant P. Mitogen-activated protein kinases (Erk1,2) phosphorylate Lys-Ser-Pro (KSP) repeats in neurofilament proteins NF-H and NF-M. J Neurosci. (1998) 18:4008–21. 10.1523/JNEUROSCI.18-11-04008.1998 9592082 PMC6792805

[64] Zhang G Kimura S Murao K Yu X Obata K Matsuyoshi H. Effects of angiotensin type I receptor blockade on the cardiac Raf/MEK/ERK cascade activated via adrenergic receptors. J Pharmacol Sci. (2010) 113:224–33. 10.1254/jphs.09336fp 20562518

