# Supplementary material for: 1,800 MHz Radiofrequency Electromagnetic Irradiation Impairs Neurite Outgrowth With a Decrease in Rap1-GTP in Primary Mouse Hippocampal Neurons and Neuro2a Cells
Source: Front Public Health. 2021 Nov 22;9:771508. doi: 10.3389/fpubh.2021.771508 (PMC8646047; doi:10.3389/fpubh.2021.771508)
Supplement: Supplementary file 1 [file Image_1.pdf]

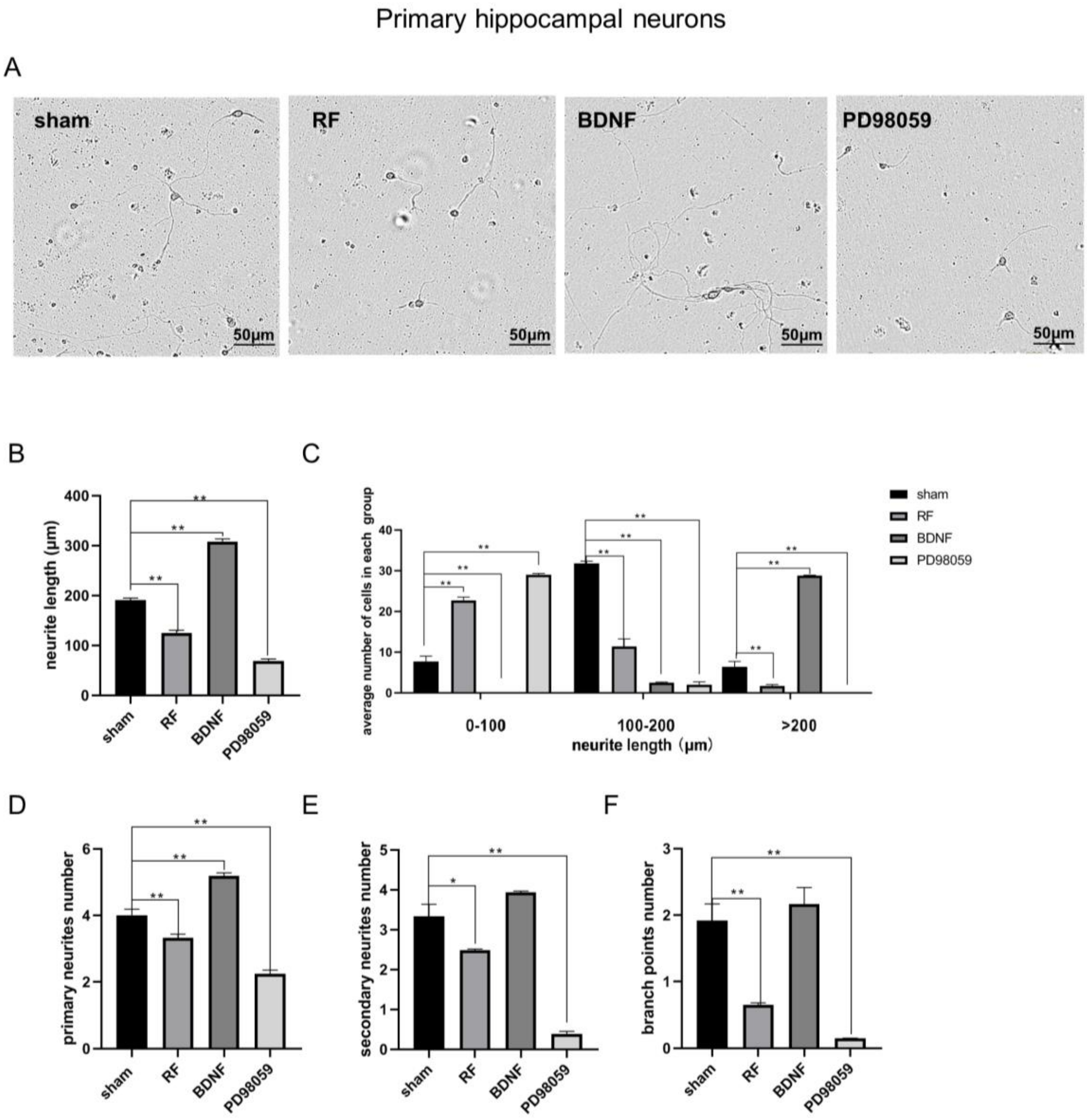

Figure S1 | Effects of BDNF (40 ng/ml, positive control) and PD98059 (50μM, negative control) on neurite outgrowth of primary hippocampal neurons.

BDNF (40 ng/ml) was incubated with primary hippocampal neurons for 48 hours as a positive control. MEK1/2 inhibitor PD98059 (50μM) was treated neurons for 48 hours as a negative control. BDNF treatment of primary hippocampal neurons could increase the average neurites length by 50% compared with sham, and could promote the number of primary neurites. However, PD98059 treatment of inhibited average neurites length by about 60%, and decreased the number of primary, secondary neurites and branch points per neuron. (A) Example images of primary hippocampal neruons were taken with a 20× optical microscope and recognized by ImageJ software. (B) shows the total neurites length per primary hippocampal neuron in the four groups. (C) shows the distribution of four groups of primary hippocampal neurons in different total length intervals of neurite outgrowth. (D,E) shows the

primary and secondary neurites number per primary hippocampal neuron of each group. (F) shows the branch points number per primary hippocampal neuron each group. * $P < 0.05$, ** $P < 0.01$, one-way ANOVA followed by Bonferroni post hoc test. Scale bar: 50 μm.

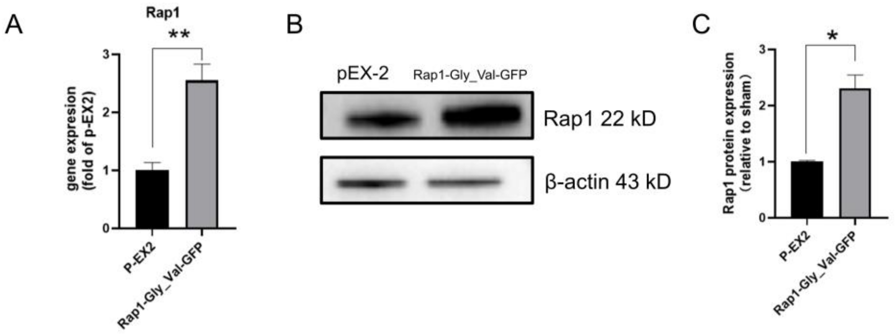

Figure S2 | Expression of Rap1 gene and protein after Rap1 constitutively active mutant plasmid transfection in Neuro2a cells.

The expression of Rap1 gene and protein in Neuro2a cells transfected with negative control plasmid and mutant plasmid (Rap1-Gly_Val-GFP) for 24 hours, Fig S2 (A-C) showed that the expression of Rap1 gene and protein in cells transfected with mutant plasmid increased. * $P < 0.05$, ** $P < 0.01$, Student's t-test.

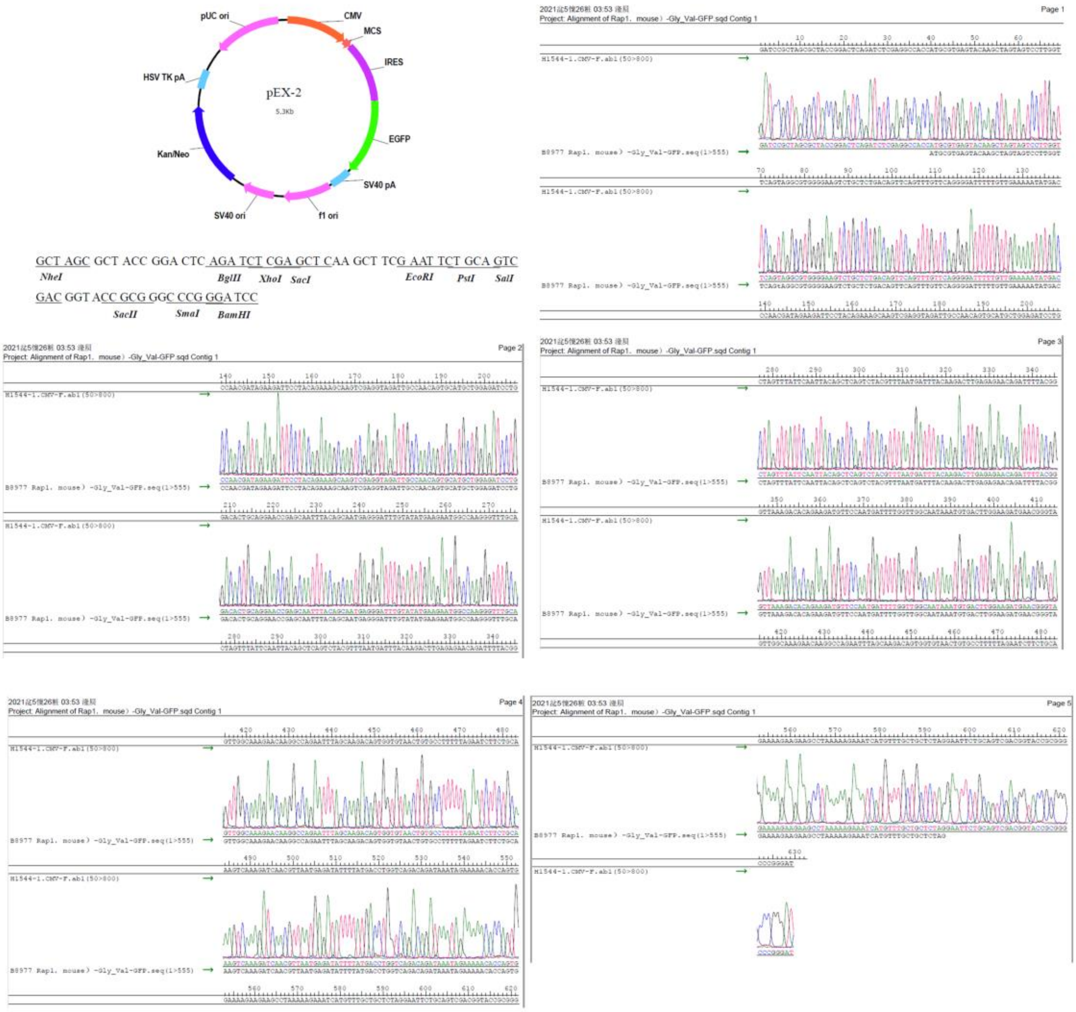

Figure S3 | Structure of Rap1 mutant plasmid.

The structure of mutant Rap1 plasmid with pEX-2 as vector provided by GenePharma company (Shanghai, China) contains the sequence of specific amino acids, the 12th amino acid of wild-type Rap1 changed from glutamic acid to valine, which could make Rap1 continuously activated.
