# Supplementary material for: 1,800 MHz Radiofrequency Electromagnetic Irradiation Impairs Neurite Outgrowth With a Decrease in Rap1-GTP in Primary Mouse Hippocampal Neurons and Neuro2a Cells
Source: Front Public Health. 2021 Nov 22;9:771508. doi: 10.3389/fpubh.2021.771508 (PMC8646047; doi:10.3389/fpubh.2021.771508)
Supplement: Supplementary file 2 [file Table_1.pdf]

Table S1 | Primer information

Gene	Forward primer (5'-3')	Reverse primer (5'-3')
Rap1a (mus)	ATGCGTGAGTACAAGCTAGTAGT	AATCTACCTCGACTTGCTTTCTG
Rap1b (mus)	GCGGGTTAAAGACACTGATGA	CTTGCTAGGTTCTGACCTTGTTC
β-actin (mus)	CGTGCGTGACATCAAAGAGAAG	CAAGAAGGAAGGCTGGAAAAGA

Table S2 | Antibody information

Antibody	Use concentration	Catalog no.	Vendor
Rabbit anti-RAP1A antibody	1： 3000	ab115776	Abcam (USA)
rabbit anti-RAP1B antibody	1： 3000	ab182606	Abcam (USA)
rabbit anti-RAP1GAP antibody	1： 3000	ab32373	Abcam (USA)
mouse anti-active Rap1 monoclonal antibody	1： 3000	26912	NewEast (USA)
rabbit anti-Rap1 antibody	1： 3000	07-916	Millipore (USA)
mouse anti-beta III Tubulin	1： 200	MAB1637	Millipore (USA)
Anti mouse Alexa fluor 555	1： 200	P0190	Beyotime (China)
mouse anti-active Rap1 monoclonal antibody	1： 200	26912	NewEast (USA)
anti-β-actin antibody	1： 5000	A1978	Sigma (USA)
Goat anti mouse secondary antibody	1： 1000	A0216	Beyotime (China)
Goat anti rabbit secondary antibody	1： 1000	A0208	Beyotime (China)
Mouse IgG antibody	1： 1000	A7028	Beyotime (China)
